# Influence of Bio-Additives on Recycled Asphalt Pavements

**DOI:** 10.3390/ma17143526

**Published:** 2024-07-16

**Authors:** Giuseppe D’Addio, Cristina Oreto, Nunzio Viscione, Rosa Veropalumbo

**Affiliations:** Department of Civil, Construction and Environmental Engineering, University of Naples Federico II, Via Claudio 21, 80125 Napoli, Italy; giuseppeuninadaddio97@gmail.com (G.D.); cristina.oreto@unina.it (C.O.); nunzio.viscione@unina.it (N.V.)

**Keywords:** asphalt mixtures, reclaimed asphalt pavement, hot recycling

## Abstract

The construction and maintenance of asphalt pavements is a resource-consuming sector, where the continuous rehabilitation of the superficial layers demands large volumes of non-renewable resources. The present work focuses on the design and characterization of asphalt mixtures for the binder layer of an asphalt pavement containing 50% reclaimed asphalt (RAP), in which seven different bio-based additives, identified as R1A, R1C, R2A, R2B, R2C, R3A, and R3B, were added to improve the workability, strength, and stiffness properties. The experimental program envisioned the hot mixing of aggregates and RAP with either a 50/70 or a 70/100 bitumen and, in turn, each of the seven bio-additives. The asphalt mixtures underwent the characterization of their densification properties; air voids; indirect tensile strength (ITS); indirect tensile stiffness modulus at 10, 20, 40, and 60 °C; and rutting resistance at 60 °C. The results highlighted that the performance in terms of workability and ITS of the resulting mixtures depends on the type of bio-additive and largely on the fresh bitumen type, while the stiffness at high temperature is not significantly affected by the presence of the bio-additives.

## 1. Introduction

The achievement of the target mechanical performance for a road superstructure presupposes an in-depth laboratory investigation on the chemical-physical system characterizing the solid-organic binder skeleton of the asphalt mixtures of which each layer of the pavement is composed, in order to return an effective and reliable solution in operation during the useful life that fully meets regulatory requirements, as well as the economic and environmental feasibility requirements [1]. The growing demand for construction and maintenance of road superstructures has involved an incessant use of natural resources with consequent negative effects in terms of environmental sustainability [2]. Many researchers are trying to identify possible solutions that lead to a minimization of the effects on the environment such as the introduction of by-products or secondary raw materials (SRM) into asphalt mixture production. For example, Tuncan et al. [3] reviewed the effects of a wide range of SRM and by-products into hot asphalt mixtures (HMAs), namely, plastic and rubber particles, fly ash, marble powder, rubber powder, and petroleum-contaminated soil as filler materials instead of mineral limestone powder into asphalt specimens. The results in terms of Marshall stability and indirect tensile strength suggested that, while the plastic, fly ash, and marble powder led to up 25% in of the mechanical performance, other solutions like the petroleum-contaminated soil had a significantly negative effect on the indirect tensile strength. Others, like Martinho et al. [4], focused on the comparison of the mechanical performance of three warm-mix asphalt (WMA) blends with recycled concrete aggregate (RCA) or electric arc furnace slag (EAFS) as substitutes of a fraction of virgin aggregates; the study evaluated the influence of introducing 60% RCA by weight or 30% of EAFS by weight into the WMA blends versus the conventional asphalt mixture. The obtained results for the WMA with by-products allowed the authors to conclude that the introduction of EAFS or RCA into the WMA blends increases the Marshall stability and may increase or decrease the resistance to rutting. The findings also showed that the stiffness modulus measured at low temperature was only slightly affected by the presence of the SRM.

One of the most common SRM that has been widely reused for the construction and maintenance of asphalt pavements is the reclaimed asphalt pavement (RAP) [5]. The RAP derives from the milling of old asphalt pavements and its reuse as partial replacement of virgin aggregate, from an environmental point of view, is allowed upon complying with limit pollutant concentrations obtained through a leaching test [6].

The large interest towards RAP reuse into asphalt mixtures is associated with improving the environmental sustainability of the construction and maintenance of asphalt pavements. For example, Chen and Wang [7] carried out a life cycle assessment study demonstrating that the use of 50% RAP into an HMA lowers the greenhouse gas production by 20% compared to a mixture without RAP. 

Similar results were found in the study by Aurangzeb et al. [8], who focused on the energy consumption of the life cycle of an HMA containing 50% RAP; the results showed that 10% reduction compared to the conventional 100% virgin HMA was achieved.

Despite the widely known and consolidated environmental benefits of using RAP into new HMAs, the mechanical aspect of introducing high amount of RAP into HMAs is still an ongoing challenge. Several studies have demonstrated that, as the quantity of RAP into HMAs increases, the indirect tensile strength and stiffness modulus sharply increase compared to a 100% virgin HMA; the embrittlement of the HMA depends on the presence of the oxidized aged bitumen inside the RAP [9].

Researchers have been working on different technical solutions to provide durable solutions with high RAP content; for example, the use of a neat bitumen with high penetration grade is one of the first and cheapest techniques applied to compensate for the aging of the bitumen contained in RAP. The purpose of this technique is to adjust the final viscosity of the binding matrix during mixing and laying of HMAs [10].

Another widespread technique is the rejuvenation of the aged bitumen contained in the RAP by using rejuvenating additives. The main objective of rejuvenators, which are mainly composed of saturated and aromatic compounds, is to dilute the oxidized and aged bitumen to reduce its asphaltene and resin content to the values typical of a first-use bitumen; therefore, the empirical and rheological performance of the resulting binder is partially restored to their original values [11].

The effectiveness and extent of the rejuvenation phenomenon strongly depends on the composition of the rejuvenating additive, as well as its dosage and the mixing characteristics of the HMA (time, temperature) [12]. In the context of time-dependent phenomena, a short-term rejuvenating effect begins with the mixing process up to the laying, compaction and opening to traffic of the asphalt pavement; this short-term phenomenon is expected to improve the handling, workability, and densification of HMAs [13]. Within this phase, the speed of diffusion of the rejuvenating additives within the oxidized bitumen depends on the properties of both the oxidized bitumen and the fresh bitumen, as well as the composition of the rejuvenating additive itself.

Different types of rejuvenating additives exist, among which the oil-based additives from organic and/or hydrocarbon origin are the most widespread in the HMA industry [14]. However, the latest trend consists of reusing waste oils such as waste lubricating oil, vegetable oil, and tall oil (a by-product of the paper production chain) [15]. The latter makes it possible to lower the viscosity of the aged bitumen and ensure easy paving of hot mixes containing the milled material [16]. The study conducted by Mumun and Al-Abdul [17] evaluated the effect of two different additives in a mass percentage equal to 7% by the weight of the RAP into an HMA containing 50% RAP: one additive was a virgin commercial one (CM) and the other derived from waste engine oil (WM). The mechanical analysis demonstrated that the WM additive was more effective in lowering the indirect tensile strength (ITS) and the indirect tensile stiffness modulus (ITSM) at 20 °C, which were lower by 18 and 22%, respectively, compared to those obtained with the conventional CM additive.

Another research study by Dalmazzo et al. [18] was carried out on three types of bituminous mixtures. The first one is a black system (BS), identified as such because it only contains RAP, namely, the aggregates coated with the oxidized black binder, without any additives. The other two HMAs are the RS-A, containing paraffinic hydrocarbon oils as an additive, and the RS-B, containing a non-toxic additive with a vegetable oil base. It should be noted that the ITS at 20 °C of the BS is equal to 0.9 MPa, while the mixtures RS-A and RS-B have, respectively, 44 and 33% lower ITS. Furthermore, in terms of stiffness modulus at 20 °C, the value measured for the BS is equal to 3850 MPa, while the RS-A and RS-B mixtures have, respectively, 1550 MPa (−59%) and 2000 MPa (−48%).

Bocci et al. [19] studied several HMAs containing bio-additives, namely, the mixtures with 0% RAP, 40% RAP, 40% RAP plus oil-based additive, and 50% RAP plus oil-based additive. While the air voids of the four mixes did not show significant variations, ranging between 2.2% and 3.1%, the ITS, on the other hand, was largely affected by the presence of the additive, which restored the ITS values to 1.35 MPa with 50% RAP and to 1.20 MPa, with 50% RAP, substantially equal to that of the mixture with 0% RAP and envisaging full rejuvenation of the oxidized binder in the RAP.

From the experimental studies present in the literature, very different and often conflicting results have been observed according to the dosage and origin of the recycled and/or bio-based oily additive, as well as to the type of bitumen, the specific layer of the pavement under study, and the tests applied to evaluate the effects of rejuvenation.

For these reasons, the aim of the present work was to evaluate a large group of oil-based additives, deriving from waste sources and eventually combined with other natural or synthetic components, to (a) identify an operative procedure to introduce the bio-additives into the asphalt mixture independently from the type and origin and (b) evaluate the effect of the bio-additives on the volumetric and mechanical performance of the resulting HMAs.

In particular, seven different oil-based recycled rejuvenating additives have been used, in turn, to blend HMAs for use in the binder layer of an asphalt pavement containing 50% RAP by weight in combination with either 50/70 or 70/100 penetration class bitumen and comparing them to the reference HMAs with 50% RAP and 0% additive. In detail, the seven additives mainly differ in terms of supplier and composition, either having a vegetable origin, eventually combined with other bio-products, resulting from the combination of a vegetable oil and one or more synthetic components, or resulting from the transformation of natural biomasses, such as pyrolysis.

The applied methods involved firstly the analysis of the workability and compactability of the designed asphalt mixtures through a volumetric and densification curve analysis and then the assessment of the effect of the temperature on the mechanical behavior in terms of indirect tensile strength at 25 °C, also considering the accumulated and dissipated energy at failure, the indirect tensile stiffness modulus in a range of service temperatures from 10 to 60 °C, and at the rutting tendency at 60 °C in full immersion in water.

## 2. Materials

### 2.1. Aggregates

**Figure 1:**
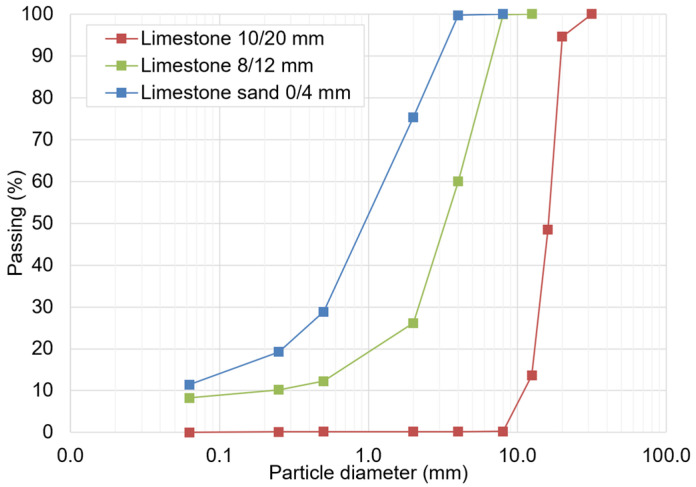
Size distributions of the limestone aggregates.

The limestone aggregates were supplied from a crushing and sieving plant located in southern Italy in 3 different sizes: limestone 10/20 mm, limestone 4/8 mm, and limestone sand 0/4 mm. The particle size distributions of each limestone aggregate size are shown in Figure 1.

The properties of these materials were evaluated in compliance with the European standard tests, whose results are shown in Table 1.

### 2.2. Reclaimed Asphalt Pavement

**Figure 2:**
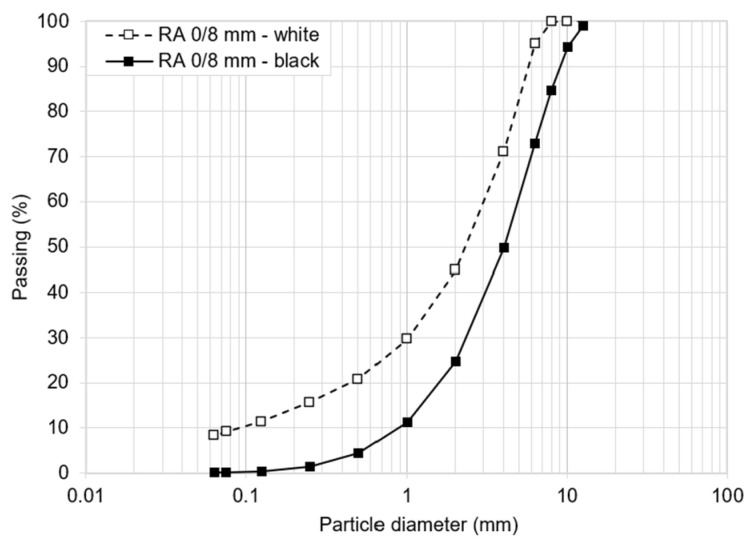
RAP size distribution.

The RAP milled from existing distressed wearing and binder layers of local asphalt pavements was reused according to the Italian regulations [6]. In detail, the screened and processed reclaimed asphalt granules were designated as RA 0/8 mm according to EN 13108-8 [26]; the main properties of the RAP aggregates and the bitumen content in the RAP are shown in Table 1. The aggregate size distributions of both the black (including the weight of the coating oxidized binder on the surface of the aggregates) and white (after the extraction of the oxidized binder) RA 0/8 mm are shown in Figure 2.

### 2.3. Bitumen

**Figure 3:**
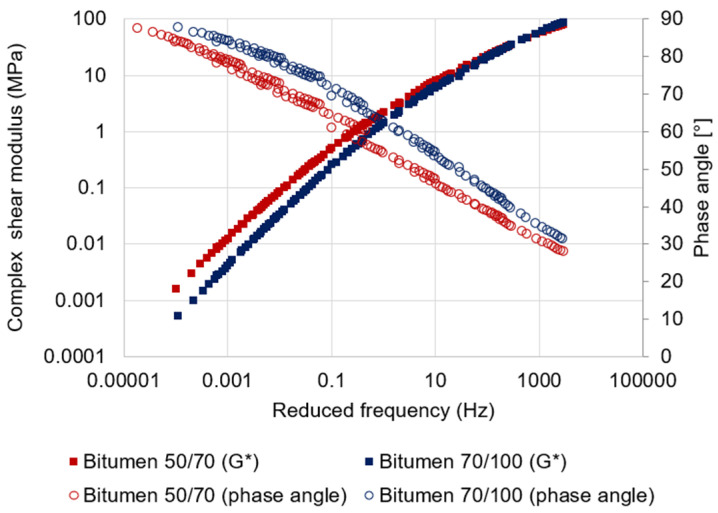
Master curves of the Bitumen 50/70 and 70/100. The solid squares represent the complex shear modulus, while the void circles represent the phase angle. The reference temperature is 20 °C.

Two different types of neat bitumen were adopted in the present study: a 50/70 and a 70/100 penetration grade (see Table 2). The frequency sweep results [27] carried out by using a dynamic shear rheometer, in the range of temperatures between 0 and 50 °C and a range of frequencies between 0.1 and 10 Hz, revealed that the greater stiffness of the bitumen 50/70 compared to bitumen 70/100 is proved by a greater shear stiffness modulus, regardless of the investigated temperatures and frequencies (see Figure 3). At the same time, the results in terms of phase angle at all test conditions showed a lower ductility of the 50/70 bitumen compared to the 70/100 bitumen.

### 2.4. Bio-Additives

Seven oily bio-additives, all having a vegetable and organic nature, were used in this study. Table 3 shows the main characteristics of the additives as provided by the suppliers. In detail, each additive, classified with its own identification code (Table 3), was introduced into the asphalt mixtures directly into the mixer to avoid procedural differences between the different mixes.

In particular, each identification code resulted from the combination of 3 characters: “R”, which stands for “rejuvenator”, a number (either 1, 2, or 3), which identifies the bio-additive producer, and a letter, which identifies the origin of the additive (either A, if the additive has a direct vegetable origin, eventually combined with other bio-products; B, if the additive results from the combination of a vegetable oil and one or more synthetic components; or C, if the additive results from the transformation of natural biomasses, such as pyrolysis).

### 2.5. Asphalt Mixture Preparation

The efficacy of bio-additives for the rejuvenation of the binder contained in the RAP was evaluated through the design of a reference system (identified as R0) composed of 50% virgin aggregates, 50% RAP, and 5% total bitumen by the weight of the aggregates, of which half derived from the oxidized binder in the RAP, and half was the fresh bitumen added, either the 50/70 or the 70/100 penetration grade bitumen. The total percentage of bitumen and the particle size distribution (see Table 4) were kept the same for all the asphalt mixtures. In this way, the effect of each bio-additive, combined with the penetration class of the bitumen, were the only variable boundary conditions of the experimental campaign, in order to investigate the effects of the physical-mechanical phenomena that occur when the oxidized bitumen film interacts with the bio-additives and the neat bitumen.

Once the reference system has been defined, the limestone aggregates in the right portion to obtain specimens with the size required by the technical standard to carry out the mechanical testing were placed in the oven for a sufficient period of time to allow the aggregates to dry and reach a suitable mixing temperature equal to 160 °C; afterwards, they were mixed together and bonded by means of bitumen, with the help of a laboratory mixer.

**Figure 4:**
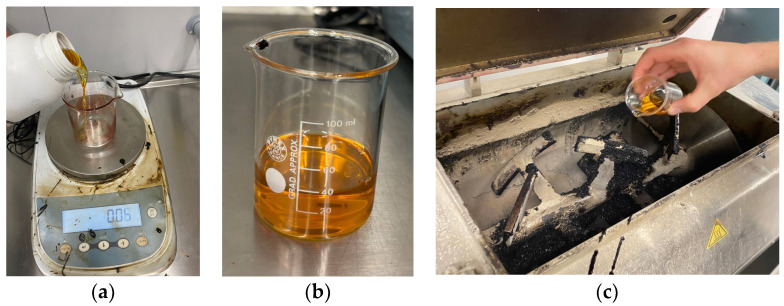
Experimental procedures: (**a**) bio-additive-dosage, (**b**) generic bio-additive appearance, and (**c**) adding the bio-additive to the horizontal asphalt mixer.

After that, the RAP was introduced directly into the mixer; mixed with the previously heated aggregates; and subsequently with the fresh bitumen and, in turn, with each bio-additive (see Figure 4).

The additives R3A and R3B were used in combination since the first had the task of improving the binder-aggregate adhesion and compactability of the mixture, while the second was specifically intended for the rejuvenation of the oxidized binder. The two additives were inserted at the same time into the mixer.

After the mixing process, which lasted 6 min to correctly coat the aggregates with bitumen, similarly to what happens in the industrial plant with a residence time equal to 60 s, the asphalt mix samples obtained were again placed in the oven, at a temperature of 150 °C, in order to avoid sharp drop in temperature. Afterwards, the asphalt mix produced was weighed (about 1300 g for each sample), placed in cylindrical stamps, and subjected to a compaction process using a gyratory compactor in compliance with the EN 12697-31 [32] standard. The initial (N_ini_), design (N_des_), and final (N_max_) number of gyrations was chosen proportional to the expected traffic expressed in equivalent standard axle loads [33], and in compliance with the tender technical specifications imposed by the main managing authorities of Italian highways (N_ini_ equal to 10, N_des_ equal to 120, and N_max_ equal to 180) [34]. Each specimen was compacted to N_max_, continuously monitoring the specimen height during the compaction process, at the end of which the specimens were extruded from the mold and allowed to cool to room temperature for 24 h.

The identification of each asphalt mixture, as well as the type of bitumen, additive, and additive dosage, is shown in Table 5.

## 3. Methods

### 3.1. Air Voids and Workability of Asphalt Mixtures

The air void content of each asphalt mixture was obtained as the average air void content measured on each specimen compacted at N_max_ according to EN 12697-8 [35], EN 12697-5 (procedure C) [36], and EN 12697-6 (procedure B) [37]. The degree of compaction achieved by the specimen at each gyration number was expressed through the %G_mm_, calculated for each number of revolutions according to Superpave manual [38].

The plot of the %G_mm_ versus the number of gyrations represents the densification curve of the asphalt mixture. The logarithmic regression of such curve is the generic function shown in Equation (1).

%G_mm_ = C + K log(n) (1)

where

- C is the self-densification;
- K the compactability;
- n is the gyration number.

### 3.2. Indirect Tensile Strength

The mechanical characterization was carried out through the investigation of the indirect tensile strength (ITS) in compliance with EN 12697-23 [39]. This type of test involves the use of cylindrical specimens loaded along the vertical diametral plane by a vertical force that develops normal tensile stresses in the horizontal diametral plane.

In addition, to evaluate the contribution of the bitumen in the determination of the ITS, the Indirect Tensile Coefficient (ITC) is calculated in compliance with Equation (2).

(2) $$ITC=\frac{\pi \;\cdot D\cdot ITS\;}{2\cdot Dc}\cdot 10\;(N/{\mathrm{mm}}^{2})$$

where

- *D* is the diameter of the specimen (mm);
- *ITS* is the indirect tensile strength (MPa);
- *Dc* is the specimen deformation, measured as the distance between the load strips at failure (mm).

Using the information provided by the ITS test, namely, the load and deformation recordings until failure, the energy accumulated and released by the specimen can be calculated.

The area under the ITS plot, before and after reaching the peak load at failure, corresponds to the work that the asphalt mixture performs to counteract the failure phenomenon. Therefore, once defined the fracture energy as the measure of the potential to resist failure, both the energy required to form a new fracture, called peak energy (E_pre-peak_), and the energy required for the propagation of the crack along the specimen diameter, called post-peak energy (E_post-peak_), as well as the total fracture energy (E_total_), were calculated using Equations (3)–(5), respectively.

(3) $$E_{pre-peak}=\frac{\int_{A}^{P}f\left(x\right)dx}{Ad}(J/m^{2})$$

(4) $$E_{post-peak}=\frac{\int_{P}^{B}f\left(x\right)dx}{Ad}(J/m^{2})$$

(5) $$E_{total}=E_{pre-peak}+E_{post-peak}(J/m^{2})$$

where

- $\int_{A}^{P}f\left(x\right)$ is the work required to trigger the crack (J);
- $\int_{P}^{B}f\left(x\right)$ is the work required to propagate the crack (J);
- Ad is the diametral surface area where the crack propagates (mm^2^).

### 3.3. Indirect Tensile Stiffness Modulus

The resilient modulus of the asphalt mixtures was measured using an indirect tensile test load configuration according to EN 12697-26—Annex C [40]. The resilient deformation was used to determine the indirect tensile stiffness modulus (ITSM) at 4 service temperatures: 10, 20, 40, and 60 °C. All the measurements were performed at a strain level of less than 50 micro-strains to be in the linear viscoelastic zone and eliminate excessive permanent deformations. The value of the ITSM was calculated in compliance with Equation (6).

(6) $$ITSM=\frac{F\cdot (\nu +0.27)}{\begin{array}{c} z\cdot h \end{array}}$$

where

- F is the peak value of the applied vertical load (N);
- z is the amplitude of the horizontal deformation obtained during the load cycle (mm);
- h is the thickness of the specimen (mm);
- ν is Poisson’s ratio, equal to 0.35.

### 3.4. Rutting

**Figure 5:**
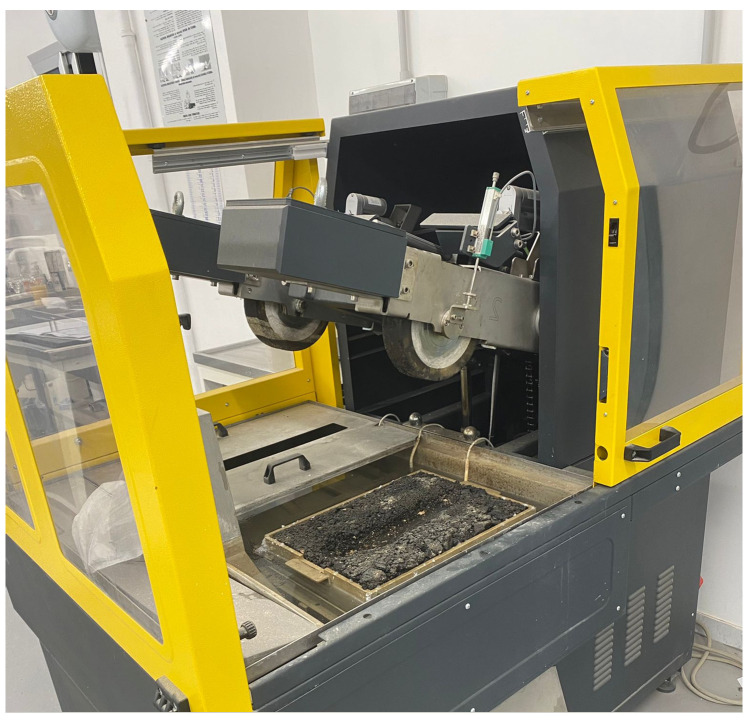
Double wheel tracking tester.

The wheel tracking test was conducted to determine the susceptibility of the asphalt mixtures containing the bio-additives to deform under load by using a wheel tracking apparatus (see Figure 5). Two slabs 30 × 40 × 6 cm for each mixture were tested in water at 60 °C, as per procedure B in EN 12697-22 [41]. The rutting sensitivity was evaluated in terms of rut depth (RD) and wheel tracking slope (WTS_water_) as the value after 10,000 cycles averaged on the two slabs, as per EN 12697-22.

## 4. Results

### 4.1. Workability

**Figure 6:**
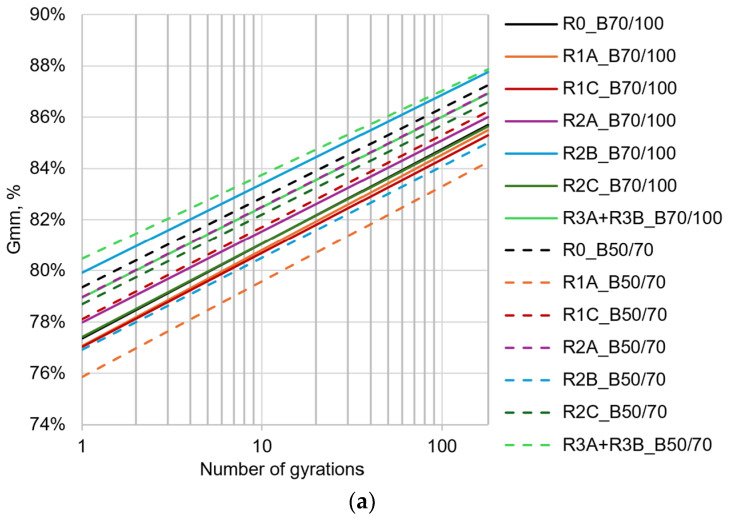

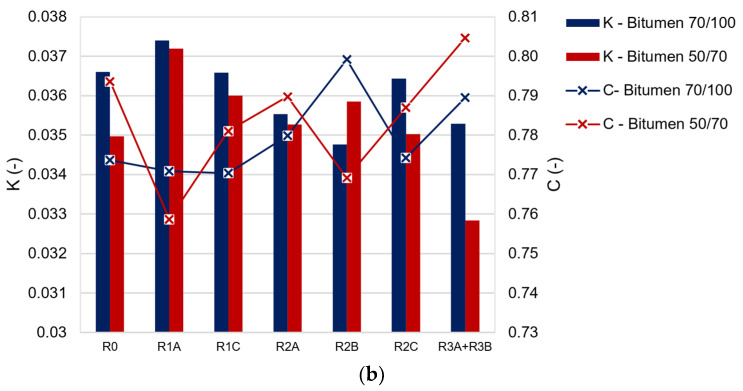
Results in terms of (**a**) densification curves and (**b**) workability parameters (self-densification “C” and compactability “K”).

The analysis of the workability was carried out in terms of self-densification (C) and compactability (K), namely, the intercept and the slope of the densification curve obtained during gyratory compaction (Figure 6a). The values of C and K for all the 14 asphalt mixtures are shown in Figure 6b.

The mixtures containing the bitumen 50/70 and the additives had, in comparison to R0_B50/70, K coefficients slightly greater by 6.4%, 3.0%, 0.9%, 2.5%, 0.2%, respectively, for R1A_B50/70, R1C_B50/70, R2A_B50/70, R2B_B50/70, R2C_B50/70, and R3A + B. On the contrary, the R3A + R3B_B50/70 revealed a 6.10% decrease in the K coefficient in comparison to R0_B50/70. Looking at the effect of the additives combined with the bitumen 70/100, only R1A_B70/100 returned a K value 1.2% higher than that of R0_B70/100; all the other mixtures, namely, R1C_B70/100, R2A_B70/100, R2B_B70/100, R2C_B70/100, and R3A + R3B_B70/100, returned a K value, respectively, 1.0%, 3.9%, 5.9%, 1.4%, and 4.5% lower than that of R0_B70/100.

These first results demonstrate that the benefits of adopting the bio-additives in increasing the compactability of the asphalt mixtures containing them varies as a function of the penetration grade of added fresh bitumen: the greater the penetration of the fresh bitumen, the lesser the effect of the bio-additives. Nevertheless, the R1A additive has proven to be the most appropriate, showing the greatest positive variation of the compactability when combined with the bitumen 50/70 and the smallest negative variation when combined with the bitumen 70/100.

The properties of self-compaction of the mixtures with bio-additives were evaluated in terms of the coefficient C of the logarithmic regression of the densification curve. Looking at the mixtures containing 50/70 neat bitumen, it is possible to observe that, in comparison to the reference asphalt mixture (R0_B50/70), all the mixtures containing bio-additives had lower C values, except for the mixture containing the R3A + R3B blend. In detail, R1A, R1C, R2A, R2B, and R2C showed C values, respectively, 4.4%, 1.6%, 0.5%, 3.1%, and 0.8% lower than that of R0_B50/70; on the contrary, the R3A + R3B_B50/70 had a C value 1.41% higher than that of R0_B50/70. These results demonstrate that, among all the analyzed bio-additives, the most effective in terms of workability is the R1A combined with the fresh bitumen 50/70.

As concerns the mixtures containing neat bitumen 70/100, different results have been observed from the previous case. In particular, as shown in Figure 6b, only R1A_B70/100 and R1C_B70/100 had C values lower by 0.4% that that of R0_B70/100; all the remaining bio-additives produced C values greater than that of R0_B70/100.

Again, as higher C values correspond to greater self-densification of the mixtures, which could lead to difficulties in handling the loose mixture during construction, the bio-additive R1A is univocally regarded as the best solution to improve the workability of the asphalt mixture.

### 4.2. Air Voids

The volumetric analysis of the 14 asphalt mixtures was carried out by evaluating the air void content in correspondence of three different number of revolutions prescribed by the technical specification tenders, namely, N_ini_ (10 revolutions), N_des_ (100 revolutions), and N_max_ (180 revolutions).

**Figure 7:**
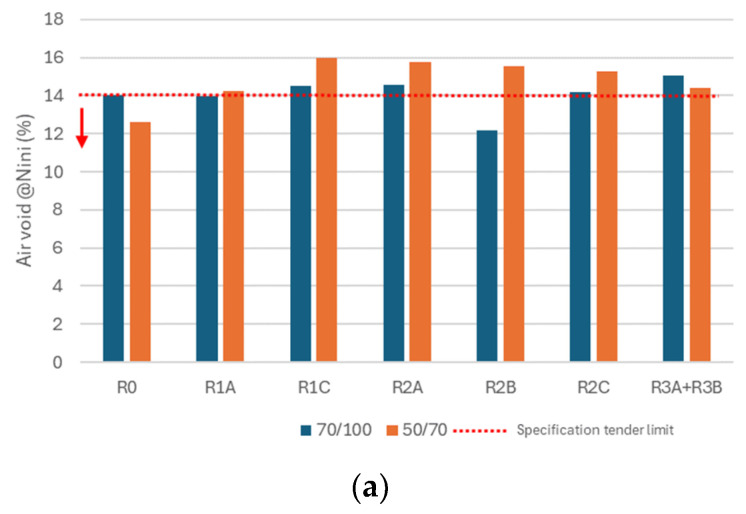

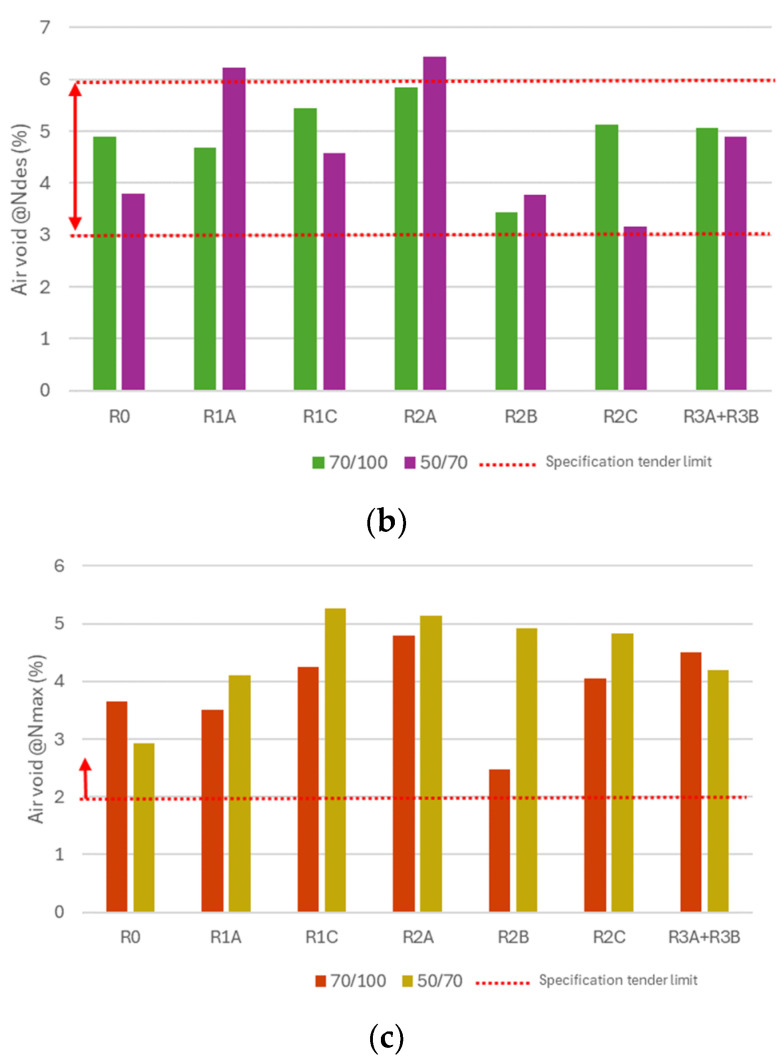
Results in terms of air void content at (**a**) N_ini_, (**b**) N_design_, (**c**) N_max_.

Looking at the air void content at N_ini_ of the mixtures containing the neat bitumen 70/100 (see Figure 7a), there was no substantial difference with the air voids of R0_70/100, except for the mixture containing the R2B additive, whose air void content was 13% lower than that of R0_B70/100.

On the contrary, the mixtures containing the bitumen 50/70 returned air void content on average 20% higher than that of R0_B50/70. The result is compliant with the observed self-densification behavior, leading to the consideration that the mixture, having more voids in this first phase of compaction, is much more workable than the reference mixture R0_B50/70.

As concerns N_des_ (see Figure 7b), the results for R1A_B70/100, R2C_B70/100, and R3A + R3B_B70/100 are very similar to that of R0_B70/100; differently, R1C_B70/100 and R2A_B70/100 returned an average increase in the air voids equal to 15% compared to R0_B70/100. The mixture containing R2B further varied compared to R0_B70/100, showing 20% air void reduction.

In the case of the neat bitumen 50/70, as the number of revolutions increased, different effects caused by the different additives emerged; the additive R2B did not produce any difference with R0_B50/70, while the additive R2A had 69% greater air voids and the additive R2C had 17% lower air voids.

At N_max_ (see Figure 7c), the additive R3B combined with the bitumen 70/100 had an air void content 32% lower than that of R0_B70/100; on the contrary, R1A did not produce any difference with respect to R0_B70/100.

In the case of the mixtures containing neat bitumen 50/70, all the additives produced an increase in the air void content compared to that of R0_B50/70, moving from an increase equal to 40% (R1A_B50/70) to 79% (R1C_B50/70).

From the results described above, it is evident that the effect of the additive changed as a function of the fresh bitumen used. It was revealed that the additive R1A did not produce significant effects within the volumetric composition of the mixtures containing the bitumen 70/100, differently from when it was used in presence of the bitumen 50/70, which produced the highest air void content compared to that of the remaining mixtures.

Although the results depend on the type of bitumen and additive inside the mixtures, all the air void content values obtained complied with, or slightly overcame, the limits imposed by the technical specification tenders. Therefore, this implies that the introduction of the additive does not produce negative variations in relation to the desired volumetric requirements, in particular when high quantities of RAP as 50% by the weight of the aggregates are reused into HMAs.

### 4.3. Mechanical Performance

#### 4.3.1. Indirect Tensile Strength

**Figure 8:**
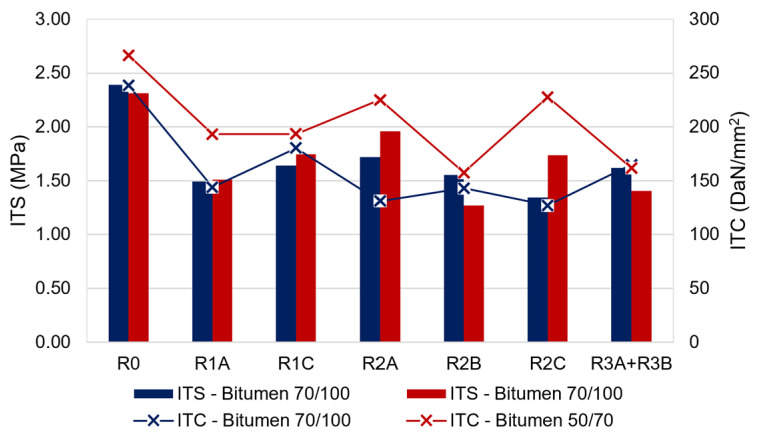
Results in terms of ITS and ITC.

As far as the evaluation of the mechanical performance of the mixtures, the first analysis was carried out by investigating the ITS at 25 °C. The results for all 14 asphalt mixtures are shown in Figure 8. It is possible to observe that all the bio-additives, both in presence of neat bitumen 50/70 and neat bitumen 70/100, produced lower values of ITS in comparison to the reference mixture R0 without additives.

In detail, the mixtures R1C_B50/70, R2A_B50/70, R2B_B50/70, R2C_B50/70, and R3A + R3B_B50/70 had on average 35% lower ITS than that of R0_B50/70; the mixture R1A_B50/70 produced the smallest reduction compared to R0_B50/70, equal to 20%.

Concerning the mixtures with neat bitumen 70/100, R1A_B70/100, R1C_B70/100, R2B_B70/100, and R3A + R3B_B70/100 had on average a 34% lower ITS value than that of R0_B70/100, while the smallest and greatest reductions, equal to -28 and -44%, respectively, were recorded for R2A_B70/100 and R2C_B70/100.

Independently from the bitumen type, the only two additives that returned quantitatively the same effect on the ITS in the presence of both the neat bitumen 50/70 and 70/100 were R2B and R3A + R3B. Furthermore, the ITS reduction was such to be compliant with the specifications, as the final measured values were above the minimum value of 0.7 MPa required for HMAs for the binder layer.

**Figure 9:**
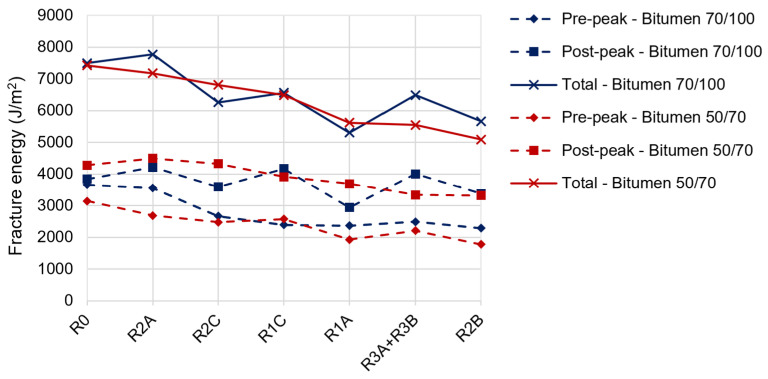
Results in terms of pre-peak, post-peak, and total fracture energy.

Further analyses were conducted on the ITS plots obtained during the ITS test. In particular, with reference to the pre-peak fracture energy of the mixtures with 70/100 bitumen (see Figure 9), all the asphalt mixtures containing the additives showed a reduction compared to R0_B70/100. In fact, the mixtures containing R1A, R1C, R2B, R2C, and R3A + R3B had a pre-peak energy value on average 40% lower than R0_B70/100, while the mixture containing R2A mixture had a fracture energy approximately equal to that of R0_B70/100. Similarly, the mixtures containing the 50/70 bitumen and the additives had a pre-peak fracture energy reduction equal to 35% compared to R0_B50/70, except for R2A_B50/70 (-15%). These behaviors are in line with the results of the ITS, where lower values have been recorded for the mixtures containing the bio-additives, which leads to stating that the mixtures with bio-additives are more deformable, are less brittle, and are therefore able to better withstand loads, delaying the crack propagation.

With reference to the post-peak fracture energy of the asphalt mixtures mixed with 70/100 bitumen (see Figure 9), it is noticeable that all the mixtures with additives resulted in greater post-peak fracture energy compared to the R0_B70/100; the exceptions was R2B_B70/100 that was equal to R0_B70/100 and R1A_B70/100 that returned 18% lower post-peak fracture energy compared to R0_B70/100.

Differently, only R2A_B50/70 and R2C_B50/70 showed higher post-peak fracture energy than that of R0_B50/70 (by 5 and 8%, respectively). The other additives (R1A, R1C, R2B, and R3A + B) produced a lower post-peak fracture energy, on average by 17%, than R0_B50/70.

Greater post-peak fracture energy values correspond to a delay in the crack propagation in asphalt mixtures; in the present case, all the additives produced this benefit in the mixtures containing 70/100 bitumen with the exception of the R1A additive, while only the R2A and R2C additives were effective when combined with the 50/70 bitumen.

#### 4.3.2. Indirect Tensile Stiffness Modulus

Table 6 shows the results of the ITSM test conducted on the asphalt mixtures under analysis at 10, 20, 40, and 60 °C. It is noticeable that, up until 40 °C test temperature, all the asphalt mixtures, regardless of the bitumen type, returned lower ITSM values compared to the reference mixtures. As concerns the mixtures with 70/100 bitumen, the reductions recorded for all the asphalt mixtures were on average equal to 21, 20, and 31% at 10, 20, and 40 °C, respectively; for the mixtures with 50/70 bitumen, the ITSM reductions were equal to 20, 40, and 28% at 10, 20, and 40 °C, respectively.

At 60 °C test temperature, an increase in the ITSM was observed for the mixtures containing the R2A with the neat bitumen 70/100 and R2B with the neat bitumen 50/70; these two mixtures, compared to the reference mixture R0, demonstrated slight ITSM increases of 6 and 4%, respectively.

These results may imply how the resistance of the mixtures at high service temperatures increases in the presence of specific additives, namely, the R2A and R2B, leading to better resistance to degradation phenomena that occur at high temperature, such as rutting.

A one-way ANOVA (analysis of variance) was performed on the ITSM data to assess whether the experimental data obtained for the mixtures containing the bio-additives were significantly different from a statistical point of view from those obtained for the mixtures without the bio-additives. In detail, a confidence level of 95% was assumed, indicating that the difference is statistically significant if the *p*-value is lower than 0.05. The results in terms of *p*-value are shown in Table 7. In detail, the ITSM variation was always significant at 10, 20, and 40 °C, while the effect of the additives was negligible at 60 °C.

Looking at the variation of the ITSM values as the test temperature increased, no substantial differences arose in terms of thermal dependency compared to the reference mixtures R0. In correspondence of both the 50/70 and 70/100 bitumen, all the ITSM values were lower on average by 39, 81, and 75% moving from 10 to 20 °C, from 20 °C to 40 °C, and from 40 to 60 °C, respectively.

#### 4.3.3. Rutting Resistance

The results obtained with the wheel-tracking device for all the asphalt mixtures under analysis are shown in Table 7. All the mixtures reached the end condition of the test (10,000 load cycles) with a ruth depth lower than 20 mm.

First of all, the trend of the RD and WTS_water_ parameters is in line with the ITSM results obtained at 60 °C test temperature. In detail, all the HMAs containing the recycled bio-additives, except for R2A_B70/100 and R2B_B50/70, had greater values of RD after 10,000 load cycles compared to the respective reference mixtures without any additives. Furthermore, all the mixtures containing the bio-additives resulted in greater values of the WTS_water_ parameter compared to the respective mixtures without additives, suggesting a greater deformation rate between 5000 and 10,000 load cycles, which may be associated with a more advanced creep stage.

Looking at the results for the mixtures with the 70/100 bitumen, the greatest RD value and worst performance was that of the mixture with R3A + R3B, followed by R1C and R2C; R1A_B70/100 and R2B_B70/100, instead, had on average 58.8% greater RD compared to that of R0_B70/100.

As concerns the mixtures with 50/70 bitumen, the rutting performance variation with respect to R0 when using the R1A additive did not depend on the bitumen type, as shown by the 51.2% increase in the RD value of R1A_B50/70 compared to that of R0_B50/70. The same goes for R1C, which produced an RD increase with respect to R0 equal to 160.3% in combination with the 50/70 bitumen, and equal to 187.1% in combination with the 70/100 bitumen. Differently, R2A and R2C achieved the worst rutting performance when combined with the 50/70 bitumen, while R3A + R3B contributed to an RD increase equal to 110.1% with respect to R0_B50/70.

## 5. Discussion

The influence of the binder type and the possible different effects of bio-additives from different sources on the compactability of asphalt mixtures was also detected by Podolsky et al. [42]; they used three different bio-additives derived from lignocellulosic biomass and two derived from pine tall oil. In particular, the use of bio-additives did not produce any differences in terms of compactability when combined with a neat PG 58-28 binder in comparison to the conventional HMA; on the contrary, the compaction index improved (40 less gyrations to reach the target air voids content, equal to 7%) when a polymer modified PG 64-28 binder was used in combination with the additives from biomass origin. The results can be considered consistent with the main findings of the present study in terms of the influence of the bio-additives on the compactability and volumetric of the HMAs: the softer the fresh bitumen, the lesser the effect of the bio-additives, independently from the waste source.

Looking at the mechanical results obtained in terms of ITS, the effectiveness of the bio-additives shifted from roughly 20% to 44% ITS reduction compared to the mixes without them. The results are consistent with the outcomes of previous studies [17,18,19] obtained using additives from different sources, like waste vegetable oil, tall oil from pine wood, and waste engine oil; in detail, for RAP content in the range 40–100% and bio-additive dosage in the range 6–13% by the RAP binder weight, the ITS reduction range was equal to 17–34%.

The ITSM of rejuvenated asphalt mixtures incorporating bio-additives was investigated by Dalmazzo et al. [18] at 20 °C and, even if the boundary conditions of the analysis (aggregate size distribution, type of fresh binder, origin of the bio-additive and RAP content) diverged from those of the present study, similar results were obtained in terms of ITSM reduction compared to that of the mixture without the bio-additives, equal to 50% for Dalmazzo et al. and to 40% in the present study for the mixtures with 50/70 bitumen.

Looking at the rutting resistance, the asphalt mixtures incorporating a significant amount of oxidized bitumen due to high RAP content (greater than 40%) often show good rutting performance: in the study by Liu et al. [43], the mixture with 70% RAP and a virgin commercial rejuvenator had a final accumulated rut after the wheel tracking test in the range 3- 6 m, similar to the outcome of the present study for the mixtures without additives and for those containing the R2A combined with the bitumen 70/100 and R2B combined with the bitumen 50/70. Nevertheless, some bio-additives in certain dosage may worsen the rutting performance compared to the no-additive condition: Im et al. [12] used 30% RAP in combination with different bio-additive dosage in the range 2–10% by weight of the total bitumen, obtaining a rut depth in the range 6–13 mm after 10,000 load cycles.

## 6. Conclusions

This study was focused on the assessment of the influence of several bio-additives, obtained from waste oily sources, on hot recycled asphalt mixtures for the binder layer of an asphalt pavement containing 50% RAP. Seven different bio-additives were examined by adding, in turn, a 50/70 and a 70/100 penetration grade bitumen as fresh binder. A total of fourteen asphalt mixtures were subject to a volumetric and mechanical investigation whose main results are as follows:

- The compactability of the asphalt mixtures containing the bio-additives varied as a function of the penetration grade of the added fresh bitumen: the greater the penetration of the fresh bitumen, the lesser the effect of the bio-additives; in addition, the R1A additive, composed of salts of ethoxylated fatty acids and waste vegetable oils, resulted as the best in terms of workability, regardless of type of added bitumen.
- It was proven that the introduction of the bio-additive, independently from the type, did not produce negative variations in relation to the desired volumetric requirements.
- All the bio-additives, both in the presence of the neat bitumen 50/70 and the neat bitumen 70/100, produced lower values of ITS at 25 °C in comparison to the reference mixture R0 without additives.
- A similar reduction trend was recorded in terms of ITSM for all the analyzed solutions containing bio-additives in comparison to the mixtures R0 at the test temperatures of 10, 20, and 40 °C; a different effect among the bio-additives was observed in correspondence of the test temperature of 60 °C, where R2A and R2B resulted in increased stiffness value compared to that of the reference mixtures. Despite the difference not being significant from a statistical point of view, a compliant result was obtained in terms of increased rutting resistance.

Further studies will be carried out to investigate the low temperature resistance of the asphalt mixtures and, in addition, economic and environmental feasibility analyses of the analyzed hot recycled asphalt mixtures will be performed, respectively, through a life cost analysis and a life cycle assessment.

## Figures and Tables

**Table 1:** Main properties of the limestone aggregates.

Properties	Unit	Standard	Limestone 10/20 mm	Limestone 8/12 mm	Limestone Sand 0/4 mm	RAP 0/8 mm
Los Angeles coefficient	%	EN 1097-2 [20]	20.6	20.1	-	19.4
Shape Index	%	EN 933-4 [21]	14	15	-	9.67
Flakiness Index	%	EN 933-3 [22]	15	16	-	18.94
Sand Equivalent	g/cm^3^	EN 933-8 [23]	-	-	85.3	-
Bulk density	g/cm^3^	EN 1097-6 [24]	2.694	2.713	2.718	2.721
Bitumen Content	%	EN 12697-1 [25]	-	-	-	5.02

**Table 2:** Bitumen properties.

Description	Standards	Units	Bitumen 70/100	Bitumen 50/70
Penetration	EN 1426 [28]	dmm	72	68
Softening point	EN 1427 [29]	°C	43.5	48.0
Viscosity @60 °C	EN 13702 [30]	Pa × s	152.44	272.46
Viscosity @100 °C	EN 13702 [30]	Pa × s	3.42	4.70
Viscosity @150 °C	EN 13702 [30]	Pa × s	0.25	0.28
*Post RTFOT*				
Penetration	EN 1426 [28]	dmm	51	58
Softening point	EN 1427 [29]	°C	50.1	56.3
Increase in the softening point	EN 12607-1 [31]	°C	6.6	8.3
Residual penetration	EN 12607-1 [31]	%	71.0	85.3

**Table 3:** Characteristics of the bio-additives.

Additive Code	Description	Dosage	Density at 25 °C (g/cm^3^)	Viscosity at 25 °C (cPs)
R1A	Liquid Oil-based Composed of salts of ethoxylated fatty acids and waste oils from vegetable origin	3.0%–5.0% by weight of the oxidized bitumen in the RAP	0.98	35
R1C	Liquid Oil-based Composed of bio-oils from pyrolysis of food waste	0.10%–0.15% by RAP weight	0.95	40
R2A	Liquid Oil-based Composed of waste oils from vegetable origin (sunflower) and natural waxes	0.05%–0.30% by RAP weight	0.92	50
R2B	Liquid Oil-based Composed of vegetable oils and plasticizers (acrylates and cellulose acetate)	0.05%–0.25% by RAP weight	0.87	70
R2C	Liquid Oil-based Composed of bio-oils from pyrolysis of food waste	0.05%–0.30% by RAP weight	0.92	50
R3A *	Liquid Oil-based Composed of	0.3%–0.6% by bitumen weight	0.90	300
R3B *	Liquid Oil-based Composed of waste oil from vegetable origin and synthetic waxes	0.1%–0.4% by RAP weight	0.87	100

* R3A and R3B are intended to be used in combination. The resulting composite additive is regarded as R3A + R3B.

**Table 4:** Composition of the reference asphalt mixture.

Component	Percentage by Weight of the Aggregates	Percentage by Weight of the Mixture
Limestone 10/20 mm	27%	25.7%
RAP (white)	50%	47.6%
Limestone sand 0/4 mm	15%	14.3%
Limestone 8/12 mm	8%	7.6%
Bitumen	5%	4.8%

**Table 5:** Composition of the asphalt mixtures.

Mixture ID	Bitumen Type	Additive Type	Additive Dosage (% by Mass of the Mix)
R0_B50/70	Bitumen 50/70	-	-
R1A_B50/70		R1A	0.095%
R1C_B50/70		R1C	0.071%
R2A_B50/70		R2A	0.071%
R2B_B50/70		R2B	0.071%
R2C_B50/70		R2C	0.071%
R3A + R3B_B50/70		R3A R3B	0.005% 0.021%
R0_B70/100	Bitumen 70/100	-	-
R1A_B70/100		R1A	0.095%
R1C_B70/100		R1C	0.071%
R2A_B70/100		R2A	0.071%
R2B_B70/100		R2B	0.071%
R2C_B70/100		R2C	0.071%
R3A + R3B_B70/100		R3A R3B	0.005% 0.021%

**Table 6:** Indirect tensile stiffness modulus results: average value, standard deviation on 3 specimen datasets, and *p*-value from one-way ANOVA versus the reference asphalt mixture without the bio-additives.

Mixture ID	Parameter	Unit	10 °C	20 °C	40 °C	60 °C
R0_B70/100	ITSM (±σ)	MPa (MPa)	15,465 (±190)	9123 (±370)	2013 (±194)	421 (±128)
R1A_B70/100	ITSM (±σ) *p*-value	MPa (MPa) -	12,773 (±249) 0.00012	7010 (±431) 0.00299	1382 (±106) 0.00787	372 (±50) 0.57728
R1C_B70/100	ITSM (±σ) *p*-value	MPa (MPa) -	11,837 (±227) 0.00029	6871 (±477) 0.00720	1446 (±92) 0.010301	339 (±51) 0.36336
R2A_B70/100	ITSM (±σ) *p*-value	MPa (MPa) -	13,370 (±403) 0.00124	8241 (±350) 0.04016	1610 (±236) 0.04968	447 (±56) 0.75674
R2B_B70/100	ITSM (±σ) *p*-value	MPa (MPa) -	12,426 (±352) 0.00067	7421 (±421) 0.00128	1452 (±155) 0.0456	380 (±38) 0.27891
R2C_B70/100	ITSM (±σ) *p*-value	MPa (MPa) -	11,262 (±514) 0.00093	6726 (±322) 0.00672	1316 (±127) 0.02669	344 (±62) 0.52383
R3A+R3B_B70/100	ITSM (±σ) *p*-value	MPa (MPa) -	11,739 (±164) 0.00037	7639 (±186) 0.00359	1183 (±166) 0.03881	313 (±44) 0.39112
R0_B50/70	ITSM (±σ)	MPa (MPa)	16,236 (±289)	9577 (±265)	2113 (±214)	441 (±39)
R1A_B50/70	ITSM (±σ) *p*-value	MPa (MPa) -	13,863 (±311) 0.00122	8788 (±320) 0.00952	1611 (±181) 0.0461	376 (±42) 0.67392
R1C_B50/70	ITSM (±σ) *p*-value	MPa (MPa) -	12,619 (±184) 0.000744	7999 (±199) 0.00341	1466 (±87) 0.02294	342 (±54) 0.61395
R2A_B50/70	ITSM (±σ) *p*-value	MPa (MPa) -	12,651 (±612) 0.00832	8628 (±294) 0.00799	1522 (±89) 0.03863	317 (±52) 0.58911
R2B_B50/70	ITSM (±σ) *p*-value	MPa (MPa) -	13,524 (±295) 0.00017	7825 (±286) 0.00612	1666 (±188) 0.02774	459 (±37) 0.49922
R2C_B50/70	ITSM (±σ) *p*-value	MPa (MPa) -	12,597 (±312) 0.00118	7980 (±139) 0.00103	1549 (±149) 0.0482	290 (±45) 0.78324
R3A+R3B_B50/70	ITSM (±σ) *p*-value	MPa (MPa) -	12,478 (±188) 0.00076	8119 (±283) 0.00741	1288 (±127) 0.00952	384 (±49) 0.64387

**Table 7:** Rutting resistance results.

Mixture ID	RD after 10,000 Cycles [mm]	WTS_water_ [mm/1000 Cycles]
R0_B70/100	4.57	0.12
R1A_B70/100	7.58	0.49
R1C_B70/100	13.12	0.30
R2A_B70/100	4.23	0.19
R2B_B70/100	6.93	0.61
R2C_B70/100	12.11	0.27
R3A+R3B_B70/100	18.12	0.86
R0_B50/70	4.16	0.15
R1A_B50/70	6.29	0.58
R1C_B50/70	10.83	0.42
R2A_B50/70	16.96	0.92
R2B_B50/70	3.12	0.18
R2C_B50/70	17.46	0.83
R3A+R3B_B50/70	8.74	0.74

## Data Availability

The original contributions presented in the study are included in the article, further inquiries can be directed to the corresponding author.

## References

[1] Santos J., Flintsch G., Ferreira A. (2017). Environmental and economic assessment of pavement construction and management practices for enhancing pavement sustainability. Resour. Conserv. Recycl..

[2] Russo F., Veropalumbo R., Oreto C. (2023). Climate change mitigation investigating asphalt pavement solutions made up of plastomeric compounds. Resour. Conserv. Recycl..

[3] Tuncan M., Tuncan A., Cetin A. (2003). The use of waste materials in asphalt concrete mixtures. Waste Manag. Res..

[4] Martinho F.C.G., Picado-Santos L.G., Capitão S.D. (2018). Influence of recycled concrete and steel slag aggregates on warm-mix asphalt properties. Constr. Build. Mater..

[5] Tarsi G., Tataranni P., Sangiorgi C. (2020). The challenges of using reclaimed asphalt pavement for new asphalt mixtures: A review. Materials.

[6] Oreto C., Russo F., Dell’Acqua G., Veropalumbo R. (2024). A comparative environmental life cycle assessment of road asphalt pavement solutions made up of artificial aggregates. Sci. Total Environ..

[7] Chen X., Wang H. (2018). Life cycle assessment of asphalt pavement recycling for greenhouse gas emission with temporal aspect. J. Clean. Prod..

[8] Aurangzeb Q., Al-Qadi I.L., Ozer H., Yang R. (2014). Hybrid life cycle assessment for asphalt mixtures with high RAP content. Resour. Conserv. Recycl..

[9] Monu K., Ransinchung G.D., Singh S. (2019). Effect of long-term ageing on properties of RAP inclusive WMA mixes. Constr. Build. Mater..

[10] Sengoz B., Oylumluoglu J. (2013). Utilization of recycled asphalt concrete with different warm mix asphalt additives prepared with different penetration grades bitumen. Constr. Build. Mater..

[11] Emtiaz M., Imtiyaz M.N., Majumder M., Idris I.I., Mazumder R., Rahaman M.M. (2023). A Comprehensive Literature Review on Polymer-Modified Asphalt Binder. CivilEng.

[12] Im S., Karki P., Zhou F. (2016). Development of new mix design method for asphalt mixtures containing RAP and rejuvenators. Constr. Build. Mater..

[13] Pradhan S.K. (2023). Short-term and long-term aging effect of the rejuvenation on RAP binder and mixes for sustainable pavement construction. Int. J. Transp. Sci. Technol..

[14] Yang S., Lee J., Hwang S., Kwon S., Baek C. (2012). Development of warm-mix asphalt additive and evaluation of performance. Transp. Res. Rec..

[15] Brownridge J. The role of an asphalt rejuvenator in pavement preservation: Use and need for asphalt rejuvenation. Proceedings of the Compendium of Papers from the First International Conference on Pavement Preservation.

[16] Gschwendt I. (2018). Extending the service life of pavements. Slovak J. Civ. Eng..

[17] Mamun A.A., Al-Abdul Wahhab H.I. (2018). Evaluation of Waste Engine Oil-Rejuvenated Asphalt Concrete Mixtures with High RAP Content. Adv. Mater. Sci. Eng..

[18] Dalmazzo D., Urbano L., Riviera P.P., Santagata E. (2022). Testing of reclaimed asphalt model systems for the evaluation of the effectiveness of rejuvenators. Proceedings of the RILEM International Symposium on Bituminous Materials: ISBM Lyon 2020.

[19] Bocci E., Cardone F., Grilli A. (2017). Mix design and volumetric analysis of hot recycled bituminous mixtures using a bio-additive. Transport Infrastructure and Systems.

[20] (2020). Tests for Mechanical and Physical Properties of Aggregates. Methods for the Determination of Resistance to Fragmentation.

[21] (2008). Tests for Geometrical Properties of Aggregates. Determination of Particle Shape. Shape Index.

[22] (2012). Tests for Geometrical Properties of Aggregates. Determination of Particle Shape. Flakiness Index.

[23] (2015). Tests for Geometrical Properties of Aggregates. Assessment of Fines. Sand Equivalent Test.

[24] (2022). Tests for Mechanical and Physical Properties of Aggregates. Determination of Particle Density and Water Absorption.

[25] (2020). Bituminous Mixtures. Test Methods. Soluble Binder Content.

[26] (2016). Bituminous Mixtures. Material Specifications. Reclaimed Asphalt.

[27] Russo F., Oreto C., Veropalumbo R. (2022). Promoting Resource Conservation in Road Flexible Pavement Using Jet Grouting and Plastic Waste as Filler. Resour. Conserv. Recycl..

[28] (2015). Bitumen and Bituminous Binders. Determination of Needle Penetration.

[29] (2015). Bitumen and Bituminous Binders. Determination of the Softening Point. Ring and Ball Method.

[30] (2018). Bitumen and Bituminous Binders. Determination of Dynamic Viscosity of Bitumen and Bituminous Binders by the Cone and Plate Method.

[31] (2014). Bitumen and Bituminous Binders. Determination of the Resistance to Hardening under Influence of Heat and Air. RTFOT Method.

[32] (2019). Bituminous Mixtures. Test Methods. Specimen Preparation by Gyratory Compactor.

[33] Southgate H.F. (1993). An Analytical Investigation of AASHTO Load Equivalencies.

[34] Viscione N., Veropalumbo R., Oreto C., Biancardo S.A., Abbondati F., Russo F. (2022). Additional procedures for characterizing the performance of recycled polymer modified asphalt mixtures. Measurement.

[35] (2018). Bituminous Mixtures. Test Methods. Determination of Void Characteristics of Bituminous Specimens.

[36] (2018). Bituminous Mixtures. Test Methods. Determination of the Maximum Density.

[37] (2020). Bituminous Mixtures. Test Methods. Determination of Bulk Density of Bituminous Specimens.

[38] Shenoy A. (2001). Refinement of the Superpave specification parameter for performance grading of asphalt. J. Transp. Eng..

[39] (2017). Bituminous Mixtures. Test Methods. Determination of the Indirect Tensile Strength of Bituminous Specimens.

[40] (2018). Bituminous Mixtures. Test Methods. Stiffness.

[41] (2023). Bituminous Mixtures. Test Methods. Wheel Tracking.

[42] Fakhri M., Norouzi M.A. (2022). Rheological And Ageing Properties of Asphalt Bio-Binders Containing Lignin and Waste Engine Oil. Constr. Build. Mater..

[43] Liu Y., Wang H., Tighe S.L., Zhao G., You Z. (2019). Effects of preheating conditions on performance and workability of hot in-place recycled asphalt mixtures. Constr. Build. Mater..

